# Interleukin-9 in Immunopathology of *Trypanosoma cruzi* Experimental Infection

**DOI:** 10.3389/fcimb.2021.756521

**Published:** 2021-10-15

**Authors:** Nadjania Saraiva de Lira Silva, Cristina Mary Orikaza, Fabiana Rodrigues de Santana, Luana Aguiar dos Santos, Bruno Ramos Salu, Maria Luiza Vilela Oliva, Rita de Cássia Sinigaglia, Renato Arruda Mortara

**Affiliations:** ^1^Microbiology, Immunology and Parasitology Department, Escola Paulista de Medicina, Federal University of São Paulo, São Paulo, Brazil; ^2^Biochemistry Department, Escola Paulista de Medicina, Federal University of São Paulo, São Paulo, Brazil; ^3^Electronic Microscopy Center, Escola Paulista de Medicina, Federal University of São Paulo, São Paulo, Brazil

**Keywords:** *Trypanosoma cruzi*, IL-9, Th9, fibrosis, collagen

## Abstract

Chagas’ disease is a parasitosis caused by *Trypanosoma cruzi*, which affects approximately 8 million people worldwide. The balance between pro- and anti-inflammatory cytokines produced during immunological responses contributes to disease prognosis and progression. Parasite tissue persistence can induce chronic inflammatory stimuli, which can cause long-term tissue injury and fibrosis. Chronic Chagas’ patients exhibit increased levels of interleukin (IL)-9, an important cytokine in the regulation of inflammatory and fibrogenic processes. Data on the role of IL-9 in other pathologies are sometimes contradictory, and few studies have explored this cytokine’s influence in Chagas’ disease pathology. Hence, the aim of this study was to evaluate the role of IL-9 in the progression of *T. cruzi* infection *in vivo* and *in vitro*. *In vitro* infection demonstrated that IL-9 reduced the number of infected cells and decreased the multiplication of intracellular amastigotes in both C2C12 myoblasts and bone marrow-derived macrophages. In myoblasts, the increased production of nitric oxide (NO) was essential for reduced parasite multiplication, whereas macrophage responses resulted in increased IL-6 and reduced TGF-β levels, indicating that parasite growth restriction mechanisms induced by IL-9 were cell-type specific. Experimental infection of BALB/c mice with *T. cruzi* trypomastigotes of the Y strain implicated a major role of IL-9 during the chronic phase, as increased Th9 and Tc9 cells were detected among splenocytes; higher levels of IL-9 in these cell populations and increased cardiac IL-9 levels were detected compared to those of uninfected mice. Moreover, rIL9 treatment decreased serum IL-12, IL-6, and IL-10 levels and cardiac TNF-α levels, possibly attempting to control the inflammatory response. IL-9 neutralization increased cardiac fibrosis, synthesis of collagens I and III, and mastocyte recruitment in BALB/c heart tissue during the chronic phase. In conclusion, our data showed that IL-9 reduced the invasion and multiplication of *T. cruzi in vitro*, in both myoblasts and macrophages, favoring disease control through cell-specific mechanisms. *In vivo*, IL-9 was elevated during experimental chronic infection in BALB/c mice, and this cytokine played a protective role in the immunopathological response during this phase by controlling cardiac fibrosis and proinflammatory cytokine production.

## 1 Introduction

Chagas’ disease is a neglected tropical disease caused by the flagellate protozoan *Trypanosoma cruzi*. Approximately 8 million people are infected worldwide, mostly in Latin America, and it causes approximately 10 thousand deaths per year. Chagas’ disease has become a global health problem as its incidence has increased in other continents, mainly due to non-vector transmission (Bastos et al., 2010; Coura and Viñas, 2010; World Health Organization, 2019).

The infection has two phases: acute and chronic. During the acute phase, there are increased numbers of parasites in the peripheral blood for up to 8 weeks, and the parasite infects various host cells such as macrophages, myocytes, and endothelial cells (Bonney et al., 2019). After a few weeks, the number of blood parasites decreases and an inflammatory immune response occurs, followed by resolution of myocardial inflammation (Bonney et al., 2019). In the chronic phase, parasitemia is low or absent, and the parasites are localized mainly in the heart and digestive muscles, which can result in cardiac, digestive, neurological or cardiodigestive clinical complications (Haberland et al., 2013; Cunha-Neto and Chevillard, 2014).

Chronic Chagas cardiomyopathy (CCC) is the most serious complication of Chagas’ disease caused by loss of function of the heart muscle and increased cardiac fibrosis, which can lead to cardiac failure and death (Haberland et al., 2013; Cunha-Neto and Chevillard, 2014). Depending on disease progression and symptoms, proinflammatory and anti-inflammatory cytokine expression can differ between patients. CCC patients have been shown to have increased IFN-γ, TNF-α, IL-6, and IL-9 levels, and reduced IL-4 and IL-10 levels compared with the levels in asymptomatic individuals (Reis et al., 1997; Poveda et al., 2014; Sousa et al., 2014). However, another study demonstrated that IL-9 levels were higher in asymptomatic individuals than in CCC patients (Guedes et al., 2016). IL-9 is a pleiotropic cytokine produced by a variety of cells, such as CD4^+^ lymphocytes of the Th9, Th2, and Th17 subsets, mast cells, eosinophils, and regulatory T lymphocytes (Goswami and Kaplan, 2011). Tc9 cells, a CD8^+^ T lymphocyte subset, also produce IL-9, which plays an important role in regulating inflammation and antitumor responses (Hoelzinger et al., 2014; Lu et al., 2014). Through its receptor (IL9R), IL-9 acts by altering signaling and protein expression in different cell types such as T and B lymphocytes, mast cells, neutrophils, macrophages, and myoblasts. IL-9 can stimulate the differentiation of CD4^+^ T lymphocytes to Th17 cells and thus inhibits *T. cruzi* intracellular multiplication in macrophages (Elyaman et al., 2009; Cai et al., 2016), otherwise IL-9 can also intensify Foxp3+ T lymphocyte functions, modulating the immune response during *T. cruzi* infection (De Araújo et al., 2011; Eller et al., 2011). Arendse et al. (2005) show that *Leishmania major* infection increases IL9-production in lymph node and splenocytes culture supernatant stimulated with promastigotes antigen at early infection stage in susceptible BALB/c mice, but not in resistant C57bl/6 mice, indicating that IL-9 acts as a susceptibility factor in leishmaniasis stimulating an adverse Th2-response in BALB/c mice.

Since IL-9 is involved in the pathogenesis of various infectious diseases in different organs, we investigated its role in *T. cruzi* strain Y infection of myoblasts and macrophages, and in a murine model of experimental infection.

## 2 Materials and Methods

### 2.1 Cells and Parasites

Murine myoblasts C2C12 cell line were cultured in RPMI 1640 medium (Vitrocell, Campinas, Brazil) supplemented with 10% fetal bovine serum (FBS) (Gibco, USA) at 37°C and 5% CO_2_. Bone marrow-derived macrophages were extracted from BALB/c mice and cultivated, as previously described (Zamboni and Rabinovitch, 2003). Tissue culture-derived trypomastigotes (TCTs) of *T. cruzi* strain Y were obtained from the supernatant of infected Vero cells and cultured in RPMI 1640 medium supplemented with 2.5% FBS at 37°C and 5% CO_2_.

### 2.2 Animals and Ethics

This study was approved by the Ethics Committee of Animal Experiments of the Federal University of São Paulo (CEUA/UNIFESP, number 8133110817). Eighty-five female BALB/c mice, aged 6–8 weeks, were maintained under standard conditions with a 12 h light-dark cycle in a temperature-controlled setting (25 ± 2°C) and *ad libitum* food and water. Euthanasia was performed according to the ethical guidelines of the Brazilian National Committee on Ethics Research (CONEP) and the National Council of Animal Experimentation Control (CONCEA).

### 2.3 Cell Viability

Cell viability was determined using an MTT (3-[4,5-dimethylthiazol-2-yl]-2,5-diphenyltetrazolium bromide) (Sigma-Aldrich, USA) assay, as previously described by Maza et al. (2008). Every *in vitro* treatment with or without infection was tested and the results were normalized to the corresponding control cell value (without treatment and infection).

### 2.4 *In Vitro* Invasion Assay

C2C12 cells or bone marrow-derived macrophages (2 × 10^4^ or 4 × 10^5^ cells/well, respectively) were seeded into 24-well plates containing round coverslips overnight at 37°C and then treated for 24 h with 1 mL of recombinant IL-9 (rIL9) (Invitrogen, USA, 25 ng/mL or 10 ng/mL, respectively) or anti-mouse IL-9 monoclonal neutralizing antibody 9CI (9CI) (BioXCell, USA, 1.25 µg/mL) diluted in RPMI medium supplemented with 10% FBS; treatment concentrations were previously established according to dose-response effect on parasite invasion and the isotype control for 9CI demonstrated identical results of control group (data not shown). TCT infection was performed with a multiplicity of infection (MOI) of 40:1 parasite:cell, and after 3 h of incubation, the non-internalized parasites were removed and the coverslips were fixed with Bouin’s solution (Merck, Darmstadt, Germany) for 15 min and then stained with Giemsa stain (Merck, Darmstadt, Germany) for 1 h. The infected cells and internalized parasites were counted in 300 cells/coverslip. Three independent experiments were performed with three technical replicates per group.

### 2.5 *In Vitro* Multiplication Assay

C2C12 or macrophages (1 × 10^4^ or 4 × 10^5^, respectively) were seeded into 24-well plates containing round coverslips overnight at 37°C. TCT infection was performed as described above. To evaluate the treatment effect only on multiplication of the intracellular parasites, the cells were treated with rIL9 or 9CI after parasites invasion (MOI 40:1), and fixed with 4% paraformaldehyde (PFA; Sigma-Aldrich) for 15 min at 48 hours post-infection (hpi), 72 hpi, and 96 hpi. After fixation, the cells were incubated with anti-*T. cruzi* monoclonal antibody mAb2C2 diluted 1:200 in PGN (0.2% gelatin, 0.1% NaN_3_, and 0.1% saponin in PBS) for 1 h at 25°C. After washing off excess mAb2C2, the coverslips were incubated with anti-mouse IgG Alexa Fluor 488 secondary antibody (Invitrogen) (1:200), DAPI (4′,6-diamidino-2-phenylindole; Invitrogen) (1:1000), and phalloidin TRITC (1:50, Sigma-Aldrich), diluted in PGN, for 1 h at room temperature. After three washes with PBS, the coverslips were mounted in glycerol buffered with 0.1 M Tris (pH 8.6) and 0.1% *p*-phenylenediamine on a glass slide. Internalized parasite numbers were observed in 100 infected cells/coverslip. Three independent experiments were performed with three technical replicates per group.

### 2.6 Parasites Released

Cells were seeded and challenged with *T. cruzi* as described in *In Vitro Multiplication Assay*. At 96 hpi, the number of released parasites in the cell medium was counted in a Neubauer chamber.

### 2.7 Nitrite Quantification

For nitrite $\left(NO_{2}^{-}\right)$ quantification of the cell medium, we used the colorimetric Griess reaction. The cells were seeded as described in *In Vitro Multiplication Assay*. Medium was collected at 72 hpi and frozen until measurement. Every plate assay included a standard curve of sodium nitrite solution ranging from 1.75–200 µM. The reaction absorbance was measured at 540 nm using a spectrophotometer.

### 2.8 Nitric Oxide (NO) Synthesis Inhibition

C2C12 cells (1 × 10^4^) were infected as described in *In Vitro Multiplication Assay*, and then 6 µM of L-NMMA (Cayman, USA), an NO synthesis inhibitor, was added to the wells. After 72 hpi, the cells were fixed with 4% PFA for 15 min and incubated with DAPI as described previously. The number of internalized parasites in 100 cells/coverslip was counted.

### 2.9 *In Vivo* Experimental Infection

Mice were inoculated intraperitoneally with 2 × 10^5^ strain Y TCTs. The infected and uninfected mice (five animals/group) were euthanized at three time points: 5 days post-infection (dpi), 9 dpi, and 90 dpi for evaluation of Th9 and Tc9 lymphocytes among the splenocytes by flow cytometry and detection of IL-9 in the heart.

To evaluate the effect of IL-9 or its neutralization, the mice were divided into five groups (five animals/group): rIL9 + infected, 9CI + infected, IgG2a (BioXCell) + infected, PBS + infected, and basal (uninfected and untreated) groups. On day -1, all mice received their respective subcutaneous treatment (9CI: 100 µg/animal, IgG2a: 100 µg/animal (Qin et al., 2016), rIL9: 50 µg/animal (De Lira Silva et al., 2019), or PBS 100µL/animal). On day 0, mice were infected intraperitoneally with 2 × 10^5^ TCTs of *T. cruzi* strain Y and were administered their respective subcutaneous treatment three times per week. After 15 dpi or 60 dpi, the mice were euthanized, blood and heart samples were collected for cytokine and histological analysis, and spleen was processed for lymphocyte population analysis.

### 2.10 Determining Th9 and Tc9 Populations

Spleen samples were ground in 5 mL RPMI 1640 medium and centrifuged at 211 ×g for 5 min at 4°C. The pelleted cells were then treated with 2 mL of hemolytic buffer (150 mM NH_4_Cl, 9 mM NaHCO_3_, and 107 μM EDTA) for 2 min. After washing to remove the lysis buffer, 5 mL RPMI 1640 medium supplemented with 10% FBS was added to the cells and clumps were removed by filtration with a cell strainer (100 μm, Corning, USA). Thereafter, viable cells (excluding trypan blue) were counted in a Neubauer chamber, and 2 × 10^6^ splenocytes were seeded into 24-well plates. Th9 and Tc9 populations were prepared as previously described (Zheng et al., 2017), and the cells were then incubated with conjugated antibodies according to the manufacturer’s recommendations (Becton Dickinson, USA): anti-CD3-PE (clone 145-2C11), anti-CD4-Pacific Blue (GK1.5), anti-CD8-PerCP-Cy 5.5 (53-6.7), anti-IL9-Alexa Fluor 647 (D9302C12), or FVS510 viability stain. Compensation beads were used for single-stain controls (OneComp eBeads, eBiosciences), and a Fluorescence Minus One (FMO) control was performed for IL-9 intracellular staining. One million events were acquired using a BD LSRFortessa flow cytometer with BD FACSDiva v.6.2 software (Becton Dickinson). Multivariate data analysis was performed using FlowJo v.9.7.6 software (Becton Dickinson).

### 2.11 Cytokine Immunoassays

The medium from infected and uninfected cells, seeded as described above, were harvested at 72 hpi. Serum and 40 µg of heart lysate were also collected from each mouse. The samples contained protease inhibitors (3 mM EDTA, Thermo Scientific, USA; 7.5 μM aprotinin, Sigma-Aldrich; and 4.6 μM E-64, Sigma-Aldrich) and were stored at -80°C until measurement. IL-12, IL-6, IFN-γ, TNF-α, IL-10, and TGF-β1 levels were measured using the MILLIPLEX^®^ MAP Mouse Cytokine kit in the MAGPIX^®^ instrument system (Merck Millipore, USA) according to the manufacturer’s instructions. Forty micrograms of heart lysates from infected (5 dpi, 9 dpi, and 90 dpi) and uninfected mice were also used to evaluate IL-9 production by MILLIPLEX^®^ MAP Mouse Cytokine kit in the MAGPIX^®^ instrument system (Merck Millipore). All samples were analyzed in duplicate.

### 2.12 Histological Analysis

Heart samples were fixed in 4% buffered formaldehyde solution, dehydrated in ethanol and xylene, and embedded in paraffin. Four-micrometer-thick sections were prepared from the paraffin blocks and were mounted on glass slides for picrosirius red (Sigma-Aldrich) or toluidine blue (Sigma-Aldrich) staining.

### 2.13 Collagen Quantification

The picrosirius red-stained slides were analyzed under a 40× objective light microscope to evaluate total collagen fibers, and quantification was performed using Image J software version 1.48t (10 fields/sample). For quantification of type I and III collagen fibers, the slides were examined under an AxioLab Standard 2.0 polarized light microscope (Carl Zeiss, Germany) for image acquisition, and the average positive pixel intensity for type I (green) and III (orange) collagen fibers in each digital image was determined with Image J software.

### 2.14 Mast Cell Quantification

The toluidine blue-stained slides were analyzed using a 100× objective light microscope to evaluate the recruitment of mast cells in the cardiac tissue. The number of degranulated or granulated mast cells and the total number of mast cells per area of the histological section were determined (number of mast cells/mm^2^).

### 2.15 Statistical Analysis

The *in vitro* assays were performed at least three times in triplicate. For the *in vivo* experiments, the data are expressed as the mean ± standard deviation of five animals per group. Significant differences were determined by one-way ANOVA, Bonferroni multiple comparison test, and Student’s t-tests (GraphPad Prism Software version 5.0). The data were considered statistically significant at p < 0.05.

## 3 Results

### 3.1 IL-9 Reduces *T. cruzi* Infection in C2C12 Myoblasts and Macrophages Through NO-Dependent and -Independent Mechanisms, Respectively

None of the stimuli used altered the cell viability, of myoblasts and macrophages at the tested concentrations (**Supplementary Figure 1**).

**Figure 1:**
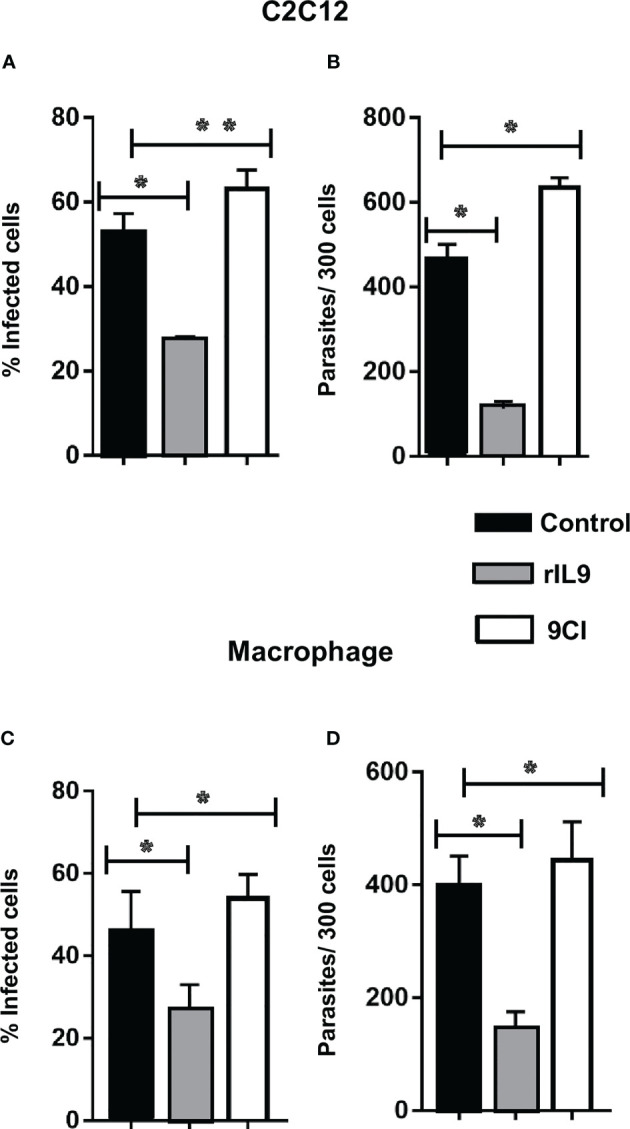
rIL9 decreased the number of infected cells and internalized parasites in C2C12 cells and macrophages, and IL-9 neutralization (9CI) reversed the effect. C2C12 cells and macrophage were pretreated for 24 h with rIL9 (25 ng/mL or 10 ng/mL) or 9CI (1.25 µg/mL) and then infected with *Trypanosoma cruzi* strain Y for 3 h. The cells were fixed with Bouin’s solution and then Giemsa stained. Graphs show the percentage of infected cells **(A, C)** and number of internalized parasites **(B, D)**. Student’s t-tests **p = 0.0172, *p < 0.001. Control: cells infected and cultured just in culture medium.

**Figure 2:**
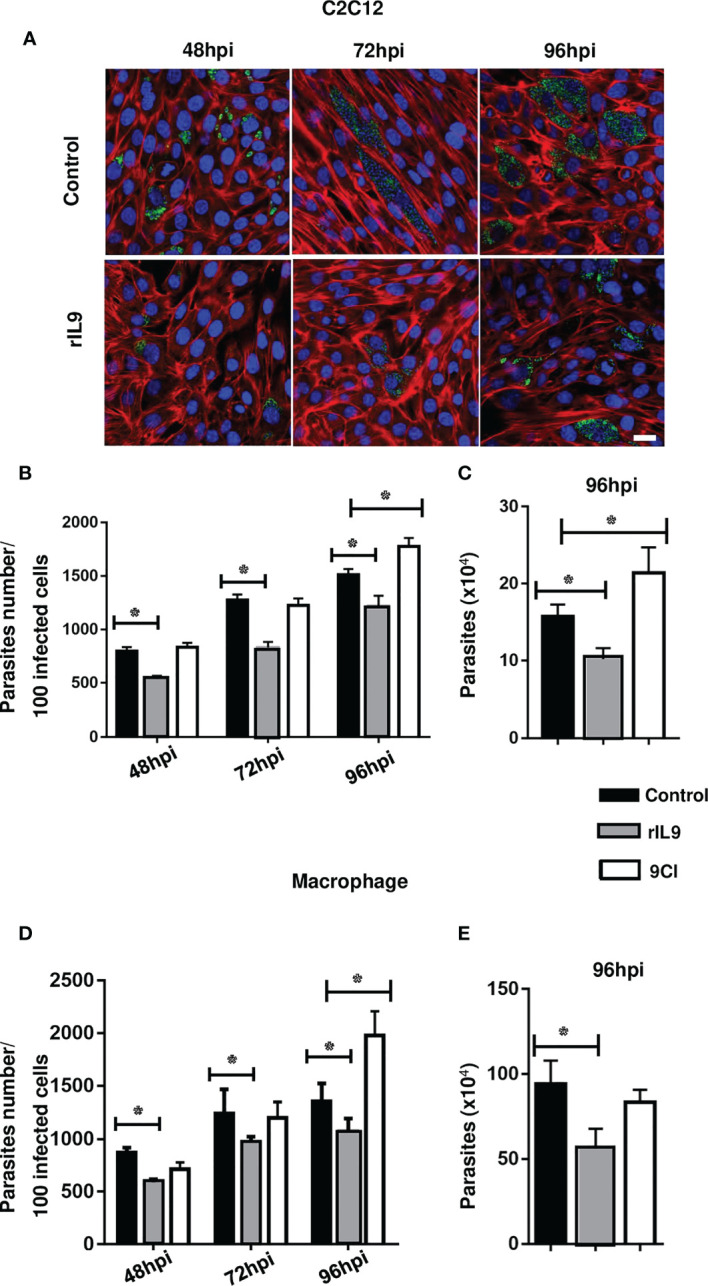
rIL9 treatment decreased intracellular amastigote multiplication in infected C2C12 cells and macrophages, and IL-9 neutralization reversed the effect. Cells were infected with trypomastigotes for 3 h and then treated with rIL9 (25 ng/mL or 10 ng/mL) or 9CI (1.25 µg/mL) for 48 hpi, 72 hpi, and 96 hpi. The following were stained: nucleus (DAPI - blue), actin cytoskeleton (TRITC - red), and parasites (mAb2C2 - Alexa Fluor 488 - green) **(A)**. Graphs show the number of parasites from 100 infected cells **(B, D)**. The numbers of parasites released after 96 hpi (infected and post-treatment of cells with rIL9 or 9CI) are shown in **(C, E)**. Control, cells infected and cultured just in RPMI medium. Anova and Student’s t-test *p < 0.01. Bar: 25 µm.

IL-9 treatment prior to cell infection with *T. cruzi* TCTs (pretreatment) significantly reduced the number of infected cells and intracellular parasites in the C2C12 myoblasts and bone marrow-derived macrophages (**Figure 1**). This reduced parasite infection was abrogated when the cells were preincubated with 9CI, a neutralizing antibody against IL-9 (**Figure 1**). To evaluate the effect of IL-9 on *T. cruzi* intracellular multiplication, the infected cells were treated with rIL9 only after parasite interaction (post-treatment). Post-treatment demonstrated that IL-9 decreased intracellular amastigote multiplication in both C2C12 myoblasts and macrophages and therefore reduced parasite release into the medium at 96 hpi (**Figure 2**). 9CI also counteracted the effect of IL-9 on intracellular parasite multiplication (**Figure 2**).

**Figure 3:**
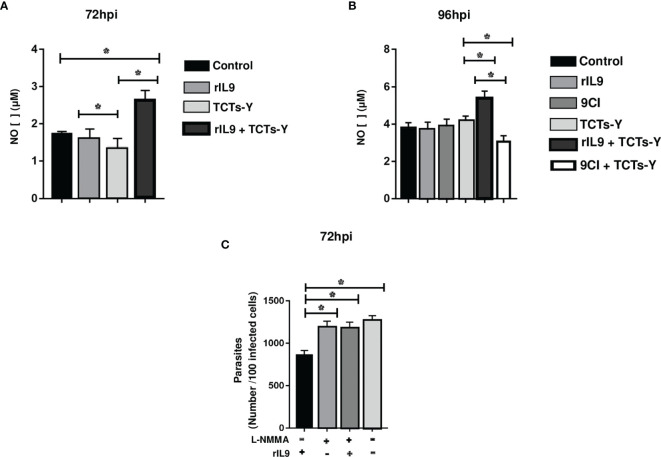
rIL9 treatment (post-treatment) increased NO production after 72 h of parasite multiplication in C2C12 cells and addition of L-NMMA inhibited the parasitic control of these cells. C2C12 cells were infected with tissue derived trypomastigotes of Y strain (TCTs-Y) for 3 h and then treated with rIL9 or 9CI. After 72 hpi **(A)** and 96 hpi **(B)**, the supernatant was collected and assayed for NO concentration using the Griess test. After 72 hpi of L-NMMA, rIL9, or L-NMMA + rIL9 treatment, infected and untreated cells were fixed with 4% PFA for 15 min and then DAPI stained to count the number of internalized parasites (in 100 infected cells) **(C)**. Anova test *p < 0.001.

NO is an important cellular molecule known to participate in *T. cruzi* killing, and our data demonstrated elevated NO levels in IL-9-treated and infected myoblasts. NO returned to basal levels with IL-9 neutralization and the inhibition of NO synthesis by L-NMMA was able to eliminate parasite growth restrictions (**Figure 3**). Although these interesting IL-9-induced NO effects were observed in C2C12 cells, the bone marrow-derived macrophages did not demonstrate alterations in NO levels in response to IL-9 or *T. cruzi* infection (**Supplementary Figure 2**), suggesting that IL-9 controls intracellular amastigote multiplication by an NO-independent mechanism in macrophages.

**Figure 4:**
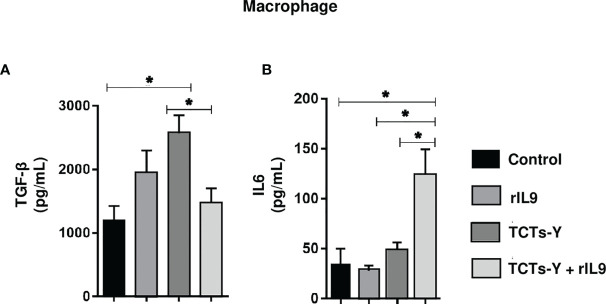
Cytokine levels in medium from infected macrophages with and without rIL9 treatment. Cell culture medium was collected at 72 hpi from C2C12 cells and macrophages cultured with RPMI medium only, cells infected and not treated, and cells infected and treated with rIL9. For infected macrophages, TGF-β secretion was lower with rIL9 treatment (post-treatment) than without treatment **(A)**, and rIL9 treatment increased IL-6 levels compared with the other evaluated groups **(B)**. Anova test *p < 0.01.

Macrophages are an important source of cytokines in response to inflammatory or infectious stimuli; indeed, *T. cruzi*-infected macrophages treated with rIL9 demonstrated reduced TGF-β and increased IL-6 levels after 72 h of infection compared with the corresponding levels in untreated infected cells (**Figure 4**). In addition, no changes in cytokine levels were observed in the C2C12 cells treated with rIL9 or in the other cytokines evaluated in macrophages (**Supplementary Figure 3**).

### 3.2 IL-9 Regulates Expression of Inflammatory Cytokines and Heart Fibrosis During the Chronic Phase of Experimental Infection by *T. cruzi*

**Figure 5:**
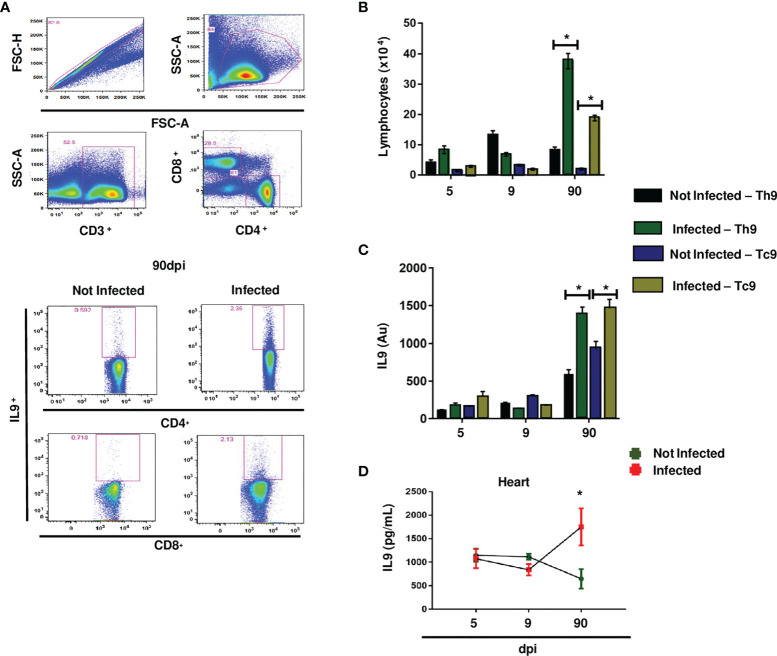
Y strain infection increased Th9 and Tc9 cells, synthesis of IL-9 in splenocytes, and cardiac IL-9 levels during chronic infection. Representative dot plots of frequency analysis of Th9 and Tc9 cells in the spleen after 90 dpi **(A)**. For selection of the population of interest, aggregates were excluded and lymphocytes were selected by size X granularity (first line of panels). Then, mature lymphocytes were selected (CD3^+^) and CD4^+^ and CD8^+^ lymphocytes were separated from them (second line of panels). CD4^+^ and CD8^+^ populations were defined as Th9 and Tc9 subpopulations (third and fourth line of panels, respectively). Absolute number of CD3^+^CD4^+^IL-9^+^ T cells and CD3^+^CD8^+^IL-9^+^ T cells **(B)**. Arbitrary units (Au) of IL-9 expression **(C)**. Quantification of IL-9 in the hearts of BALB/c mice **(D)**. Anova test *p < 0.01.

No significant differences in IL-9 levels or IL-9-secreting cells (evaluated according the gating strategy on **Figure 5A**) were detected in the acute phase of infection. However, during chronic infection, we found increased numbers of Th9 and Tc9 cells (**Figure 5B**) and elevated synthesis of IL-9 in these cell populations (**Figure 5C**). Furthermore, increased production of IL-9 was detected in the hearts of infected mice (**Figure 5D**).

Since IL-9 production was demonstrated to be higher during the chronic phase of *T. cruzi* model infection, we investigated the role of this cytokine in the immunopathological response to parasite infection. We treated a group of mice with IL-9-neutralizing antibody (9CI) or rIL9 and evaluated cytokine levels, splenocyte populations, histological heart infiltrates, and heart fibrosis during acute and chronic infection phases (15 dpi and 60 dpi, respectively). As expected, IL-9 neutralization or treatment with rIL9 did not alter any cytokine levels in the serum or heart of the infected mice at 15 dpi (**Supplementary Figures 4**, **5**).

**Figure 6:**
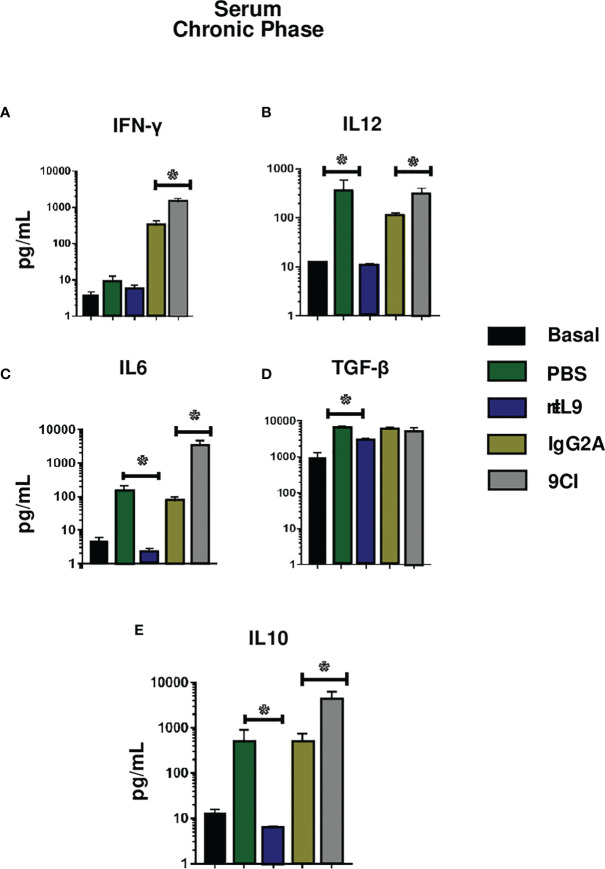
Cytokine levels in serum from infected BALB/c mice, those treated with 9CI or rIL9, and the respective control groups. Cytokines were quantified in serum from infected mice, those treated with 9CI or rIL9, and control mice (IgG2a and PBS, respectively) after 60 dpi (chronic phase). IL-9 neutralization stimulated IFN-γ **(A)**, IL-12 **(B)** IL-6 **(C)**, and IL-10 **(E)** synthesis relative to the control group (IgG2a) during chronic infection. rIL9 treatment reduced IL-6 **(C)**, IL-12 **(B)**, TGF-β **(D)**, and IL-10 **(E)** production. Anova test *p < 0.001. Basal: uninfected and untreated mice.

**Figure 7:**
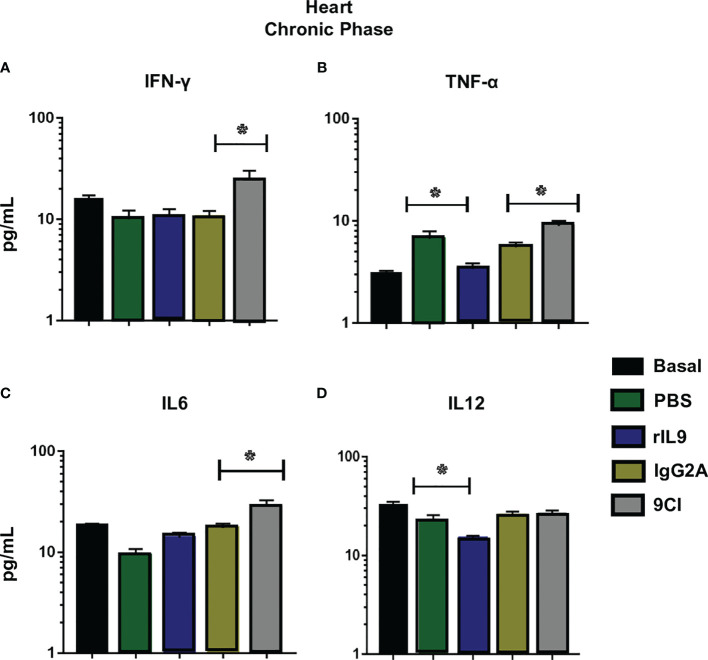
Cytokine levels in heart lysates from infected BALB/c mice, those treated with 9CI or rIL9, and the respective controls. Cytokines were quantified in heart lysates (40 μg of protein) from infected mice, those treated with 9CI or rIL9, and control mice (IgG2a and PBS, respectively) after 60 dpi (chronic phase). IL-9 neutralization stimulated IFN-γ **(A)**, TNF-α **(B)**, and IL-6 **(C)** synthesis during chronic infection. rIL9 treatment reduced IL-12 **(D)** production Anova test *p < 0.001. Basal: uninfected and untreated mice.

However, at 60 dpi, IL-9 neutralization resulted in significantly higher levels of IFN-γ, IL-12, IL-6, and IL-10 (**Figure 6**), and increased production of IFN-γ, IL-6, and TNF-α in cardiac tissue compared to the corresponding levels in anti-IgG2a-treated mice (**Figure 7**). In contrast, rIL9 treatment decreased IL-6, IL-10, IL-12, and TGF-β serum levels (**Figure 6**), and TNF-α and IL-12 cardiac levels during the chronic phase (**Figure 7**). The other evaluated cytokines showed no significant differences in their levels (**Supplementary Figures 4**, **5**).

**Figure 8:**
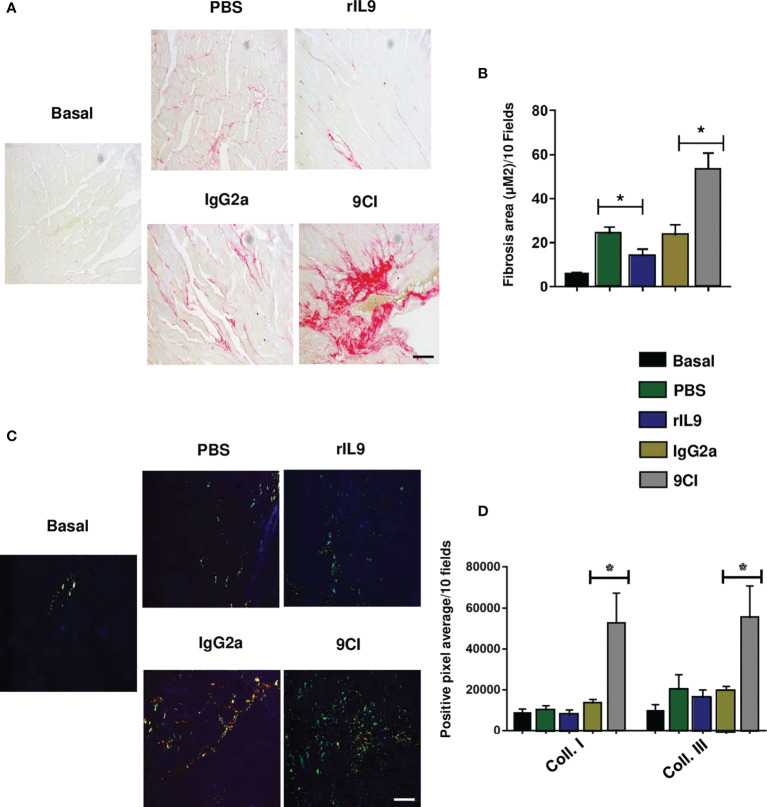
IL-9 neutralization increased and rIL9 treatment reduced cardiac fibrosis in *Trypanosoma cruzi*-infected mice after 60 dpi. **(A)** Representative photomicrograph of cardiac fibrosis after 60 dpi in uninfected and untreated mice (basal), and those infected and treated with: PBS, rIL9 (50 ng/animal), 9CI (100 μg/animal), or IgG2a (100 μg/animal). Black arrow shows the collagen network stained with picrosirius red. **(B)** Graph shows the quantification of total collagen fibers (fibrosis area) in 10 random fields. **(C)** Representative photomicrograph of type I collagen fibers (red), type III collagen fibers (green), and both fiber types (yellowish/orange) in cardiac tissue from uninfected and untreated (basal), infected and treated with PBS, infected and treated with rIL9 (50 ng/animal), infected and treated with 9CI (100 μg/animal), or infected and treated with IgG2a (100 μg/animal) mice after 60 dpi. **(D)** Graph shows the quantification of type I and III collagen fiber in 10 random fields (microscope objective 40×). Anova test *p < 0.001. Control: uninfected and untreated mice. Bar: 500 μM.

To understand the impact of IL-9 neutralization and treatment with rIL9 in the development of cardiac fibrosis in *T. cruzi-*infected mice during the chronic stage, we quantified the total collagen area and type I and III collagen levels in cardiac tissue. IL-9 neutralization significantly increased total collagen production compared to that in controls (**Figures 8A, B****)**. Moreover, rIL9-treated mice showed a reduction in total collagen area compared with PBS-treated mice, but no difference in type I or III collagen production (**Figures 8C, D****)**.

**Figure 9:**
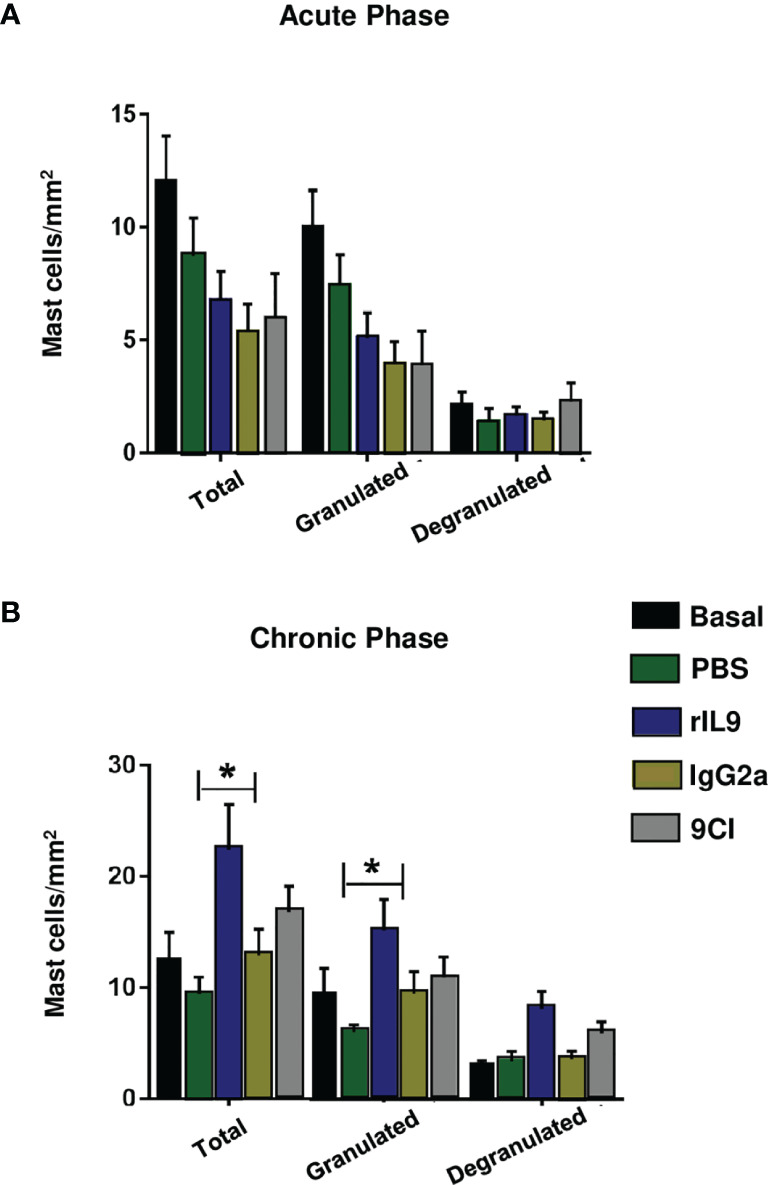
rIL9 treatment increased the recruitment of total mast cells, mostly granulated mast cells, in cardiac tissue from *Trypanosoma cruzi*-infected mice after 60 dpi. Total numbers of mast cells, granulated mast cells, and degranulated mast cells were counted in cardiac tissue from *T. cruzi*-infected BALB/c mice, those treated with 9CI or rIL9, and the respective control mice (treated with IgG2a and PBS, respectively). The mice were euthanized after 15 dpi (acute phase) **(A)** and 60 dpi (chronic phase) **(B)**. Control: uninfected and untreated mice. Anova test *p < 0.001.

Next, we investigated the possible relationship between cardiac fibrosis and mast cell recruitment in the heart. The mast cell count was significantly higher in rIL9-treated mice than in the control mice during the chronic phase (**Figure 9**); most of the cells were granulated. IL-9 neutralization did not change mast cell recruitment into the cardiac tissue (**Figure 9**).

## 4 Discussion

IL-9 is a pleiotropic cytokine mainly produced by Th9 and Tc9 cells; it participates in allergic or tumoral processes and autoimmunity responses (Licona-Limón et al., 2017). Some studies have shown the role of Th9/IL-9 in different parasitic infections, mainly involving the immune response against helminths (Tuxun et al., 2015; Licona-Limón et al., 2017). Data about the role of IL-9 in other pathologies are scarce and sometimes contradictory, and few studies have explored the influence of this cytokine on Chagas’ disease pathology.

In experimental studies, IL-9 acts in the signaling of diverse cells such as macrophages, myoblasts, and T and B lymphocytes through its receptor IL9Rαγ (Alvarez et al., 2002; Goswami and Kaplan, 2011). Our *in vitro* results showed that rIL9 treatment of *T. cruzi*-infected myoblasts and macrophages reduced invasion and intracellular amastigote multiplication. A previous experimental study indicated that IL-9 inhibited Coxsackievirus B3 viral replication and diminished the myocarditis induced by infection; these authors suggested that IL-9 reduced the levels of a host cell protein used by the virus to enter the cells (Yu et al., 2016).

We observed that IL-9-induced NO acts reducing parasite multiplication in myoblasts, although IL-9 treated cells could not abrogate intracellular amastigote growth, thus, NO seems to be an auxiliary microbicidal mechanism assisting the parasite multiplication control by C2C12 cells. Indeed, other studies in the literature support the role of NO in *T. cruzi* multiplication control, and its trypanocidal action on infected myocytes (Balligand et al., 1994; Machado et al., 2000). Many studies have shown that nitrite induced by *T. cruzi* infection can downmodulate PGE2 and COX-2, and these molecules may inhibit parasite invasion and tissue damage of heart muscle cells (Borges et al., 1998; Pinge-Filho et al., 1999; Malvezi et al., 2014; Guerrero et al., 2015; Lovo-Martins et al., 2018), so this may be an intracellular pathway triggered by IL-9 in C2C12 myoblast cells, but further studies are required to investigate this mechanism. Regarding infected and uninfected macrophages, no significant difference in NO levels was detected among groups, and this phenomenon can be partially explained by the fact that BALB/c-naive macrophages produce only small amounts of NO (Mills et al., 2000).

In the present study, we observed that rIL9 treatment of infected macrophages reduced TGF-β and increased IL-6 levels, resulting in parasitic control. TGF-β production is associated with the invasion and survival of parasites in cells, as well as with myocardial fibrosis (Waghabi et al., 2002). IL-6 is a proinflammatory cytokine that acts as an antiparasitic agent against *T. cruzi* during the acute phase, but in the chronic phase, it is related to tissue damage (Gao and Pereira, 2002; Zanluqui et al., 2020). Interestingly, chronic phase also demonstrated elevated IL-6 serum levels in response to infection and also in heart lysates when neutralizing IL-9, probably these cytokines can interplay a fine tune between beneficial inflammation and tissue damage throughout Chagas’ disease.

Recently, it has been argued that *T. cruzi* survival is related to the ability of the parasite to remain dormant under stressful conditions, like benznidazole treatment or inflammatory environment (Sánchez-Valdéz et al., 2018; Martins et al., 2020). Some intracellular amastigotes may decrease or interrupt their replication cycle and remain inconspicuous to the immune system or intracellular killing mechanisms and this dormancy state is reversible, thus, under favorable conditions, the parasite can return to its normal replicative state (Sánchez-Valdéz et al., 2018; Resende et al., 2020). Maybe IL-9 effect on parasite multiplication led to a stressful condition and, despite the decreased number of infected cells and parasites, these mechanisms were not completely effective in killing intracellular parasites, but just inducing amastigotes to enter a dormant state, later reversed when convenient.

We observed that myoblasts and macrophages responded differently to IL-9, and the different invasion mechanisms used by *T. cruzi* to invade phagocytic and non-phagocytic cells may interfere with the effect of this interleukin (Souza et al., 2010). IL-9 was effective in controlling the multiplication of replicative intracellular amastigotes, but was probably ineffective against dormant amastigotes. One possibility is that IL-9 can control parasite replication by stimulating NO synthesis (C2C12) or IL-6 secretion (macrophages) that contribute to a stressful environment, consequently favoring intracellular amastigotes to enter and/or remain in a non-replicative dormant state. Thus, the TCTs released into the extracellular medium probably originated from the reversed dormant state of amastigotes.

Regarding the role of IL-9 *in vivo*, Guedes et al. (2016) observed that patients with indeterminate Chagas’ disease exhibited higher levels of IL-9 in peripheral blood than did cardiac patients. However, Poveda et al. (2014) demonstrated that patients with cardiomyopathy showed increased IL-9 expression in the blood when compared to individuals without CCC. Guedes et al. (2016) and Poveda et al. (2014) showed that IL-9 participates in the chronic phase of Chagas’ disease, although there are population (Colombians without comorbidities versus Brazilians with or without comorbidities) and methodology (cytokine measurement by flow cytometry versus mRNA quantification, respectively) differences between the studies. However, the association of IL-9 with protective or cardiomyopathy-promoting effects in Chagas’ disease was not conclusive. Similarly, we demonstrated that infection with Y strain *T. cruzi* induced an increase in IL-9 production by Th9 and Tc9 cells after 90 dpi (chronic phase) in BALB/c murine splenocytes, but in our infection model we could demonstrate an important role of IL-9 in host protective response to control parasite load *in vitro* and also to diminish cardiac fibrosis *in vivo*.

CCC is associated with excessive inflammation and persistent immune system activation with a local increase in different proinflammatory cytokines by T lymphocyte and mononuclear cell infiltrates in the heart tissue (Cunha-Neto et al., 2011). The mechanism by which the parasite stimulates IL-9 production is unknown, but it could be related to parasite antigen recognition by pathogen-associated receptors. Parasitic antigens stimulate IFN-γ and IL-10 production, activating TLR2, which participates in the differentiation of Th9 cells derived from CD4^+^ T lymphocytes (Cardillo et al., 1996; Karim et al., 2017). IL-4 and TGF-β synthesis during the chronic phase potentiate the generation of IL-9-producing cells (Soares et al., 2001; Waghabi et al., 2002; Goswami and Kaplan, 2011).

A previous study by our research group showed that G strain *T. cruzi* infections induced early IL-9 production in the serum of BALB/c mice, however, mice that were infected with the CL strain showed increased IL-9 levels at the peak of parasitemia; with either strain, the levels returned to baseline during chronic infection (Ferreira et al., 2018). Furthermore, no change in IL-9 synthesis was observed at any of the evaluated time points (2 dpi, 8 dpi, and 90 dpi) in the serum from CL or G strain-infected C57BL/6 mice (Ferreira et al., 2018). However, Rodrigues et al. (2016) reported that C57BL/6 mice orally infected with the G or CL strain showed increased IL-9 expression in the heart during chronic infection. The results mentioned above demonstrate how the parasite’s genetic variability, infective form, and inoculation pathway are decisive factors in the parasite-host interaction and consequent development of the response against infection, disease outcome, and treatment efficacy.

New functional subtypes of Th and Tc lymphocytes have been described, such as Th9 and Tc9 lymphocytes. However, no studies to date have described their roles and importance in the context of *T. cruzi* infection. For the first time, we present data demonstrating increased splenic Th9 and Tc9 lymphocytes, both large IL-9-producing populations during chronic infection by the Y strain. Patients in the chronic phase of Chagas’ disease show an increase in activated T lymphocyte frequency and these cells secrete proinflammatory and anti-inflammatory cytokines (Dutra et al., 2009). CD8^+^ T lymphocytes mediate protection against infection by secreting cytokines such as IFN-γ and TNF; however, chronic stimulation is involved in the inflammatory process of Chagas’ disease (Sathler-Avelar et al., 2012). CD4^+^ T cells are important for generating an immune response against the parasite, and the low frequency of these IFN-γ-producing cells with *T. cruzi* infections is associated with the severity of cardiomyopathy in patients (Acevedo et al., 2018).

rIL9 treatment reduced cardiac fibrosis but did not alter collagen I and III synthesis. These results show that IL-9 is an important cytokine that acts along with the response to infection. The effect of IL-9 is evident in the chronic infection stage of the Y strain in our model, demonstrated mainly by the control of cardiac fibrosis.

IL-9 neutralization intensifies cardiac fibrosis during chronic infection, concomitantly with an increase in the systemic circulation and local synthesis of proinflammatory cytokines (TNF-α, IFN-γ, IL-6, and IL-12) that are related to cardiac damage (Satoh et al., 1999; Kassiri et al., 2005; Sun et al., 2007; Rodríguez-Ângulo et al., 2017). IL-9 reduces TNF-α levels in the heart, and the latter cytokine is linked to cell signaling cascades that modulate the host’s defense against injury, promote apoptosis, increase metalloproteinase expression (MMP), and induce tissue fibrosis (Sun et al., 2004; Connolly et al., 2009; Chaves et al., 2019). Increased TNF-α expression is associated with the development of several cardiac diseases, such as myocardial infarction, ventricular remodeling, and CCC (Satoh et al., 1999; Kassiri et al., 2005; Sun et al., 2007). Rodrigues et al. (2012) showed a positive correlation between cardiac damage and cardiac fibrosis in biopsies from patients who died after CCC complications. TNF-α induces cardiomyocyte apoptosis and activates nitric oxide synthase 2 (NOS2) to produce NO, which contributes to tissue damage during chronic infection in Chagas’ disease (Finkel et al., 1992; Tostes et al., 2005).

Chagas disease patients with ventricular dysfunction show an increase in TNF-α, IFN-γ, IL-12, IL-6, and IL-10 plasma levels, which act as important biomarkers of heart disease and suggest an association between the synthesis of Th1 and Th2 profile cytokines in serum and local cardiac inflammation (López et al., 2006; Rodríguez-Ângulo et al., 2017). Some studies have correlated IFN-γ and IL-10 synthesis with severe cardiac deficiency (Bahia-Oliveira et al., 1998; Corrêa-Oliveira et al., 1999). These results corroborate our data since IL-9 neutralization, in addition to increasing the synthesis of proinflammatory cytokines, intensified IL-10 production in the serum. Meléndez et al. (2010) showed that the pathogenic increase in circulating IL-6 levels in hypertensive rats resulted in extensive cardiac fibrosis. Endogenous IL-6 in the presence of soluble IL-6 receptor (sIL6R) increases collagen synthesis in fibroblast cultures, in addition to stimulating the differentiation of fibroblasts into myofibroblasts that act in the development of tissue fibrosis (Meléndez et al., 2010). Some studies have shown that the increase in IL-6 synthesis during chronic infection by *T. cruzi* is related to the development of chronic inflammation and cardiac fibrosis (Fontes et al., 2015; Ayala et al., 2016). According to López et al. (2006), IL-6 is strongly associated with cardiac damage progression and the symptomatic chronic phase of Chagas’ disease.

TGF-β is another cytokine that has a strong profibrotic property and contributes to cardiac damage in several fibrotic diseases (Dobaczewski et al., 2011). This cytokine is related to Chagas’ disease pathophysiology and acts in different stages of disease progression (Araújo-Jorge et al., 2012). Chagas disease patients who develop severe heart disease demonstrate high levels of circulating TGF-β (Pérez et al., 2011; Araújo-Jorge et al., 2012). These data are in agreement with our results since our infected rIL9-treated group showed reduced fibrosis and decreased TGF-β serum levels at 60 dpi.

With respect to mast cell recruitment in cardiac tissue, we observed that rIL9 treatment increased the total number of mast cells during chronic infection, and most of these were granulated cells. IL-9 neutralization did not alter mast cell recruitment, which may be explained by the fact that the absence of IL-9 activity maintains mast cells at baseline levels (Goswami and Kaplan, 2011; Sismanopoulos et al., 2012). Endogenous IL-9 increases the expression of vascular endothelial growth factor (VEGF) and IL-13; however, it does not induce degranulation or release of other mediators such as TNF-α (Sismanopoulos et al., 2012; Tete et al., 2012). VEGF and IL-13 are molecules related to the antifibrotic role of mast cells. In the presence of apoptotic neutrophils, IL-13 stimulates the polarization of macrophages to the M2 profile and reduces the expression of proinflammatory cytokines associated with decreased tissue fibrosis (Allakhverdi et al., 2007; Bosurgi et al., 2017; Legere et al., 2019). VEGF promotes angiogenesis and capillarization of cardiac tissue, a process that contributes to reduced tissue fibrosis (Legere et al., 2019). We hypothesize that rIL9 treatment stimulates the recruitment of mast cells to the heart and induces the secretion of VEGF and IL-13, which helps in extracellular matrix remodeling and consequent reductions in cardiac fibrosis.

Thus, our results demonstrate that rIL9 treatment controls *T. cruzi* infection in myoblasts and macrophages *via* different mechanisms. IL-9 possessed important activity in the control of intracellular parasitic load and chronic infection by the NO synthesis pathway in myoblasts and the regulation of inflammatory balance through IL-6 increases and TGF-β decreases. *In vivo*, Y strain TCTs stimulated the production of IL-9 in the heart and Th9 and Tc9 splenic cells in the chronic phase of Chagas’ disease. We believe that increased IL-9 production during chronic infection in *T. cruzi*-infected BALB/c mice contributes to the control of local inflammatory responses and acts to balance the inflammation related to CCC development.

## Data Availability Statement

The raw data supporting the conclusions of this article will be made available by the authors, without undue reservation.

## Ethics Statement

The animal study was reviewed and approved by Ethics Committee of Animal Experiments of the Federal University of São Paulo (CEUA/UNIFESP, number 8133110817).

## Author Contributions

NS, CO, and RM conceived the study. NS, CO, and RM designed the experiments. NS, CO, LS, MO, RS, FS, and BS performed the experiments. NS, CO, and RM interpreted the results. NS, CO, and RM wrote the manuscript. All authors contributed to the article and approved the submitted version.

## Funding

This work was supported by Fundação de Amparo à Pesquisa do Estado de São Paulo (FAPESP) (2016/15000-4 and 2017/17103-8), Coordenação de Aperfeiçoamento Pessoal de Nível Superior (CAPES), and Conselho Nacional de Desenvolvimento Científico e Tecnológico (CNPq).

## Conflict of Interest

The authors declare that the research was conducted in the absence of any commercial or financial relationships that could be construed as a potential conflict of interest.

## Publisher’s Note

All claims expressed in this article are solely those of the authors and do not necessarily represent those of their affiliated organizations, or those of the publisher, the editors and the reviewers. Any product that may be evaluated in this article, or claim that may be made by its manufacturer, is not guaranteed or endorsed by the publisher.
