# Supplementary material for: Interleukin-9 in Immunopathology of Trypanosoma cruzi Experimental Infection
Source: Front Cell Infect Microbiol. 2021 Oct 15;11:756521. doi: 10.3389/fcimb.2021.756521 (PMC8554238; doi:10.3389/fcimb.2021.756521)
Supplement: Supplementary file 1 [file DataSheet_1.docx]

**Supplementary Data**

**
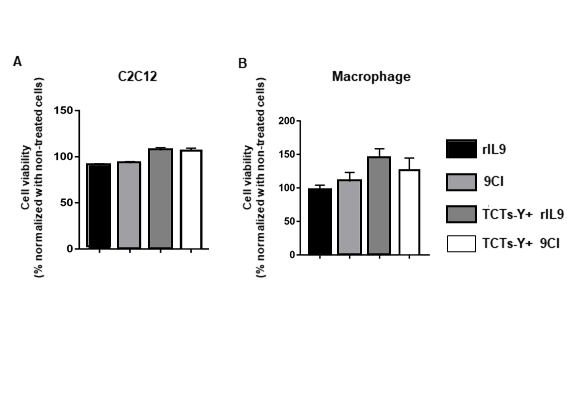
**

**Supplementary Fig. 1: rIL9 or 9CI did not affect cell viability after 72 hours of treatment in C2C12 cells or macrophages.** C2C12 cells or bone marrow-derived macrophages were infected with tissue-derived trypomastigotes (TCTs) of Y strain *Trypanosoma cruzi*, and after 3 h of incubation, non-internalized parasites were removed and respective groups were treated with recombinant IL-9 (rIL9) (25 ng/mL and 10 ng/mL, respectively) or IL-9-neutralizing antibody 9CI (1.25 µg/mL). After 72 hours post treatment, cell viability was determined using MTT.

**
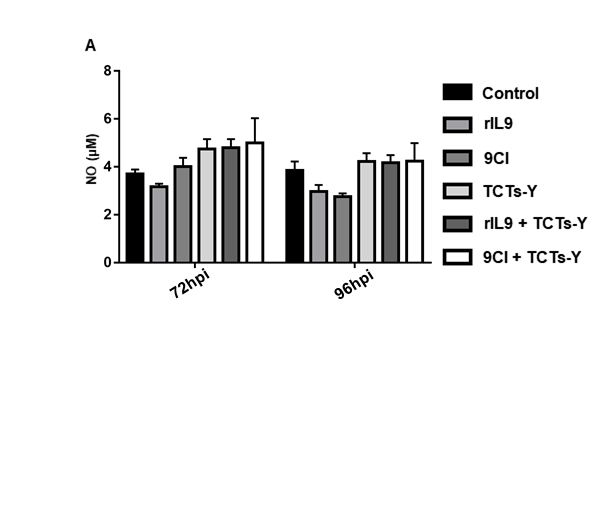
**

**Supplementary Fig. 2: rIL9 or 9CI treatment of macrophages did not interfere with NO production.** Bone marrow-derived macrophages were infected with tissue-derived trypomastigotes (TCTs) of Y strain *Trypanosoma cruzi*, and after 3 h of incubation, non-internalized parasites were removed, and respective groups were treated with recombinant IL-9 (rIL9) (10 ng/mL) or IL-9-neutralizing antibody 9CI (1.25 µg/mL). After 72 hours post-infection (hpi) and 96 hpi, medium was collected and NO levels determined by the Griess reaction. Control group represents uninfected cultured cells.

**
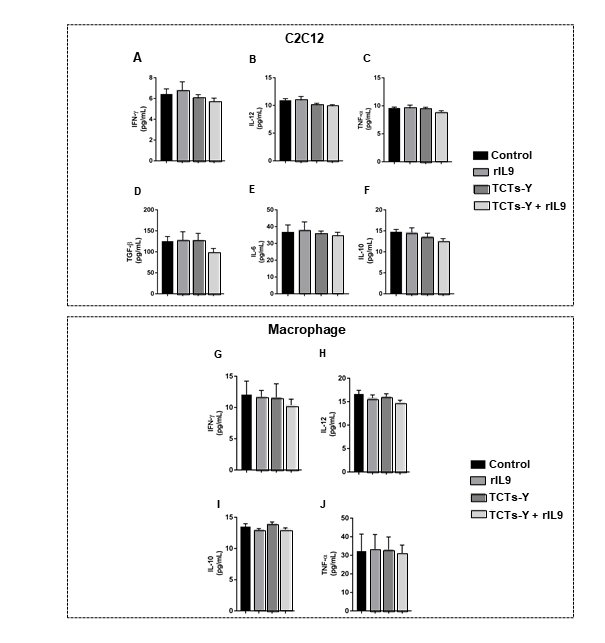
**

**Supplementary Fig. 3: Cytokine levels in medium from cultured C2C12 cells and macrophages**. C2C12 cells or bone marrow-derived macrophages were infected with tissue-derived trypomastigotes (TCTs) of Y strain *Trypanosoma cruzi*, and after 3 h of incubation, non-internalized parasites were removed, and respective groups were treated with recombinant IL-9 (rIL9) (25 ng/mL and 10 ng/mL, respectively) or IL-9-neutralizing antibody 9CI (1.25 µg/mL). After 72 hours post-infection (hpi), cell medium was collected and cytokines measured with a magnetic bead assay. No significant differences were observed among the groups.


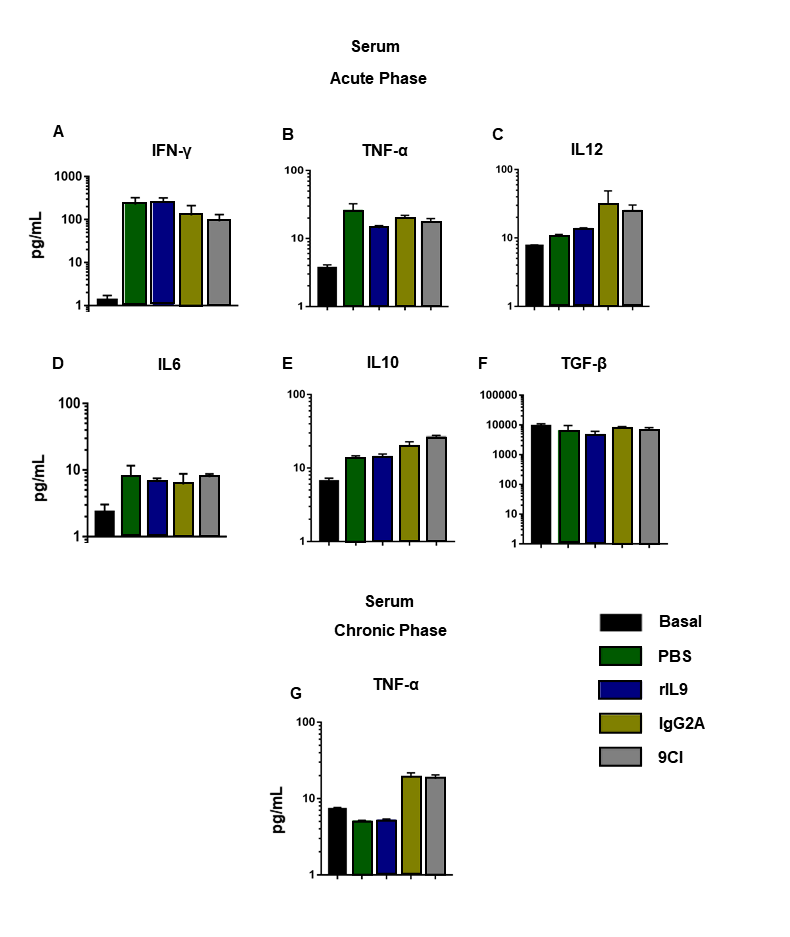


**Supplementary Fig. 4: Cytokine levels in serum from infected BALB/c mice treated with 9CI or rIL9.** Cytokines were quantified with magnetic beads in serum (A–G) from infected mice that were treated with 9CI (100 µg/animal) or rIL9 (50 ng/animal), and those treated with IgG2a and PBS, their respective control groups. The mice were euthanized and serum analyzed after 15 dpi – acute infection (A–F) or 60 dpi – chronic infection (G). There was no significant difference in cytokine level compared to that of the corresponding control. Basal: uninfected and untreated mice.


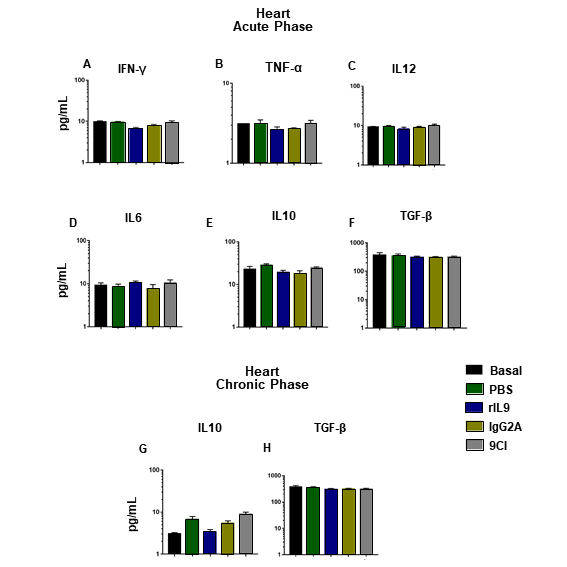


**Supplementary Fig. 5: Cytokine levels in heart lysates from infected BALB/c mice treated with 9CI or rIL9.** Cytokines were quantified with magnetic beads in 40 μg of heart lysates (A–H) from infected mice that were treated with 9CI (100 µg/animal) or rIL9 (50 ng/animal), and those treated with IgG2a and PBS, their respective control groups. The mice were euthanized and heart lysates analyzed after 15 dpi – acute infection (A–F) or 60 dpi – chronic infection (G and H). There was no significant difference in cytokine level compared to that of the corresponding control. Basal: uninfected and untreated mice.
